# Single-Step Direct Hydrothermal Growth of NiMoO_4_ Nanostructured Thin Film on Stainless Steel for Supercapacitor Electrodes

**DOI:** 10.3390/nano8080563

**Published:** 2018-07-24

**Authors:** V. Kannan, Hyun-Jung Kim, Hyun-Chang Park, Hyun-Seok Kim

**Affiliations:** 1 Millimeter-Wave Innovation Technology Research Center (MINT), Dongguk University-Seoul, Seoul 04620, Korea; kannan@dongguk.edu; 2 Division of Electronics and Electrical Engineering, Dongguk University-Seoul, Seoul 04620, Korea; best7hj@daum.net (H.-J.K.); hcpark@dongguk.edu (H.-C.P.)

**Keywords:** NiMoO_4_, nanostructures, hydrothermal, supercapacitor, stainless steel

## Abstract

We report a facile and direct growth of NiMoO_4_ nanostructures on a nonreactive stainless steel substrate using a single-step hydrothermal method and investigated hydrothermal growth duration effects on morphology and electrochemical characteristics. The highest specific capacitances of 341, 619, and 281 F/g were observed for NiMoO_4_ with 9, 18, and 27 h growth, respectively, at 1 A/g. Thus, grown samples preserved almost 59% of maximum specific capacitance at a high current density of 10 A/g. All samples exhibited a respectable cycling stability over 3000 charge-discharge operations. NiMoO_4_ grown for 18 h exhibited 7200 W/kg peak power density at 14 Wh/kg energy density. Thus, the proposed single-step hydrothermal growth is a promising route to obtain NiMoO_4_ nanostructures and other metal oxide electrodes for supercapacitor applications.

## 1. Introduction

Supercapacitors have been widely studied as candidates for energy storage systems due to their large power density, rapid charge-discharge, excellent rate capabilities, and good endurance [1,2,3]. They have been used in tandem with rechargeable storage devices and fuel cells, and they have been broadly applied for electric vehicles, electrical grid buffers, space applications, and memory system power back-up. In the recent past, there has been significant surge of research in transition metal oxides-based supercapacitors due to their multiple oxidation states and reversible redox reaction capabilities [4,5,6]. However, progress in supercapacitor electrode materials has been constrained by high cost (e.g., RuO_2_) or environmental impact (e.g., metal sulfides) [7,8,9].

Metal oxides with two transition metals have also been studied to take advantage of their variable oxidation states, excellent electrical conduction, and enhanced pseudo-capacitance characteristics, such as CoMoO_4_ [10,11], NiCo_2_O_4_ [6], ZnWO_4_ [12], and NiMoO_4_ [13]. NiMoO_4_ is a promising binary metal oxide due to its potential for high specific capacitance arising from the excellent electrochemical nature of the Ni ion, although practical electrode use is deterred by its poor electrical conductivity. Intricate characteristics of the molybdate also complicate NiMoO_4_ nanostructure growth [14,15]. Hence, it is important to identify easier preparation routes for NiMoO_4_ nanostructure thin films with distinct morphologies and superior supercapacitor characteristics. 

There is a promising route to grow the nanostructures directly on conducting substrates [16], countering inherent low metal oxide electrical conductivity and, hence, considerably enhancing electrochemical performance. There are many reports of NiMoO_4_ supercapacitor performance on Ni foam substrates [14,15,16,17,18]. However, the NiMoO_4_ on Ni foam (usually coated with a binder as a slurry) is often dominated by the Ni foam contribution to overall electrochemical performance, making it difficult to identify the exact supercapacitance value of the desired electrode material. Only NiMoO_4_ nanostructure synthesis on nonreactive substrates has been reported previously, such as stainless steel (SS) and carbon [16,19], without exploring its electrochemical characteristics. However, there are no reports available detailing the electrochemical performance of NiMoO_4_ thin film nanostructures on nonreactive substrate such as stainless steel. Here we intend to exclusively evaluate the electrochemical performance of NiMoO_4_ thin film nanostructures, without the hindrance of substrate contribution.

This study reports on the binder-free growth of NiMoO_4_ nanostructures on SS substrate using a facile, single-step hydrothermal technique. The optimal growth time was identified by studying NiMoO_4_ thin films grown for 9, 18, and 27 h, keeping other deposition conditions constant. Nanostructured NiMoO_4_ grown on SS substrate exhibited high specific capacitance, good cycling stability, and enhanced rate capability. The proposed technique offers a promising, environmentally friendly, and relatively low cost direct route to obtain high supercapacitance NiMoO_4_ nanostructures. 

## 2. Materials and Methods

### 2.1. NiMoO_4_ Nanostructure Growth Process

Molybdenum chloride (1.5 mmol) was mixed with methanol (50 mL) and stirred for 10 min. Nickel chloride solution (1.5 mmol) was added and stirred for a further 10 min, then EDTA (1.5 mmol) was added and stirred for 1 h. After forming a clear solution, 1 mL H_2_O_2_ and 1 mL HNO_3_ were added and stirred for 10 min. The solution was transferred to a Teflon container that already contained a pre-cleaned SS substrate with 1 cm × 2 cm exposed area. The complete setup was placed in a stainless steel autoclave at 180 °C. In order to identify optimal growth time, experiments were conducted with wide ranging growth durations from 9 to 36 h. Three samples were identified with 9, 18, and 27 h growth duration and labeled as NMO-9, NMO-18, and NMO-27, respectively, due to their superior electrochemical performance over the other samples. The grown films are then harvested, washed with deionized water, and dried with N_2_ gas.

### 2.2. Materials Characterization

The obtained thin films were characterized using a field emission scanning electron microscope (FE-SEM, Hitachi-S-4800, Huntington Beach, CA, USA), transmission electron microscope (TEM, JEM-2100F, JEOL, Akishima, Tokyo, Japan), and high angle annular dark field imaging (HAADF) scanning transmission electron microscope (STEM, JEM-2100F, JEOL, Akishima, Tokyo, Japan). Electrochemical measurements were performed in a 2 M KOH aqueous solution using a standard three-electrode electrochemical cell in Versa-stat-3. NiMoO_4_ served as the working electrode, with a saturated calomel electrode (SCE) and graphite rod as reference and counter electrodes, respectively.

## 3. Results

**Figure 1:**
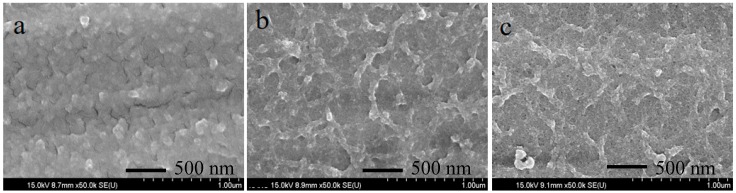
Typical field emission scanning electron microscope (FE-SEM) images for (**a**) NMO-9; (**b**) NMO-18; and (**c**) NMO-27 samples.

**Figure 2:**
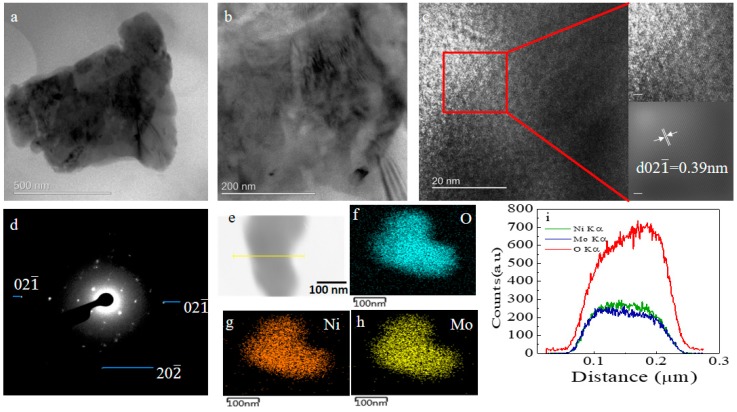
The transmission electron microscope (TEM) image for a single NiMoO_4_ nanograin grown for 18 h at (**a**) low and (**b**) high magnification; (**c**) The high resolution transmission electron microscope (HRTEM) image with d spacing noted; (**d**) The selected area diffraction (SAED) pattern of the same sample showing respective planes. High angle annular dark field imaging scanning transmission electron microscope (HAADF-STEM) elemental mapping for a selected area showing (**e**) the electron with line mapping; (**f**) oxygen; (**g**) nickel; (**h**) molybdenum; and (**i**) line mapping, showing the counts of Mo, Ni, and O.

FE-SEM morphology of the grown films for the durations of 9 h, 18 h, and 27 h are shown in Figure 1. NiMoO_4_ nanostructures are evident for all the films grown for different durations, exhibiting a stacked structure with NiMoO_4_ nanograins. TEM analyses were carried out only on sample NMO-18 due to its superior electrochemical performance in comparison with NMO-9 and NMO-27. Figure 2a,b show typical TEM images for the 18 h sample, revealing crystalline NiMoO_4_ grain-like structure. From the observed twist dislocations in Figure 2b, it can be inferred that the crystals are composed of narrow platelets. Figure 2c shows a typical high resolution transmission electron microscope (HRTEM) image from a single nanograin. The interplanar distance (0.39 nm) corresponds to monoclinic NiMoO_4_ (02$\bar{1}$) crystal planes (JCPDS: 45-0142). Figure 2d shows the selected area diffraction (SAED) pattern, which confirms the polycrystalline nature. Figure 2e–h shows the HAADF-STEM electron, O, Ni, and Mo mappings, respectively, using the same scale is the TEM images. Ni, Mo, and O are relatively uniformly distributed, which is confirmed by the elemental line mapping, as shown in Figure 2i. 

**Figure 3:**
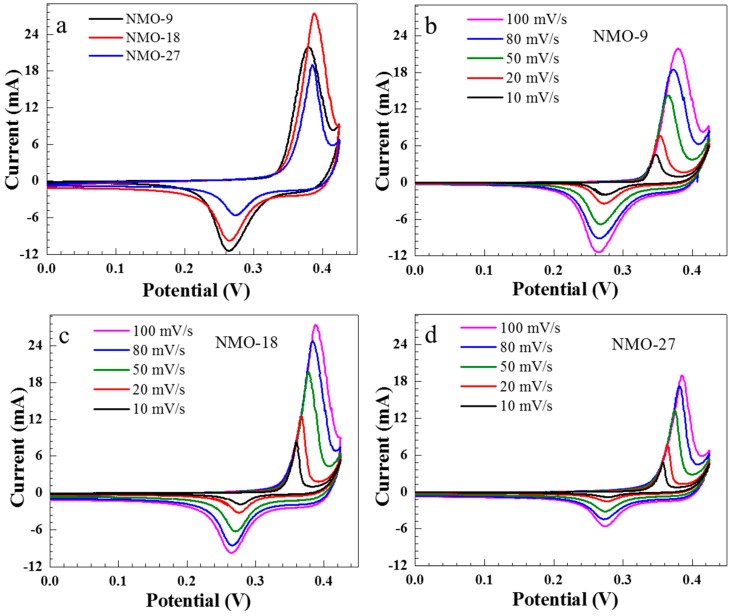
Cyclic voltammograms (CVs) for (**a**) NMO-9, NMO-18, and NMO-27 samples at 100 mV/s scan rate and various scan rates for (**b**) NMO-9; (**c**) NMO-18; and (**d**) NMO-27.

The electrochemical characteristics of the electrode are mainly dependent on their dimensions and morphologies [20,21]. Electrochemical measurements of NiMoO_4_ nanostructure were performed in 2 M KOH electrolyte. Figure 3a shows the cyclic voltammograms (CV) for hydrothermally grown NiMoO_4_ at different durations (100 mV/s scan rate), over the potential range of 0–0.43 V (versus SCE). Each CV plot exhibits distinct redox peaks, indicating that the observed capacitance properties can be described by Faradic reactions [22,23]. The NMO-18 sample had the largest area under the CV curve compared with NMO-9 and NMO-27.

Figure 3b–d show NMO-9, NMO-18, and NMO-27 CVs, respectively, for various scan rates. The CV curve shapes are almost unchanged for the different scan rates, indicating near ideal capacitive characteristics. Voltage corresponding to the oxidation peak moved in the positive direction as the scan rate increased, whereas the reduction peak moved in the negative direction, which can be attributed to electrode’s internal resistance [24].

The surface area accessible for electrochemical reactions can be estimated from the electrochemically active surface area (ECSA). Initially, the non-Faradic capacitive current was obtained from the linear region of the CV curves,

*i*_DL_ = *C*_DL_ × *ν* (1)

where *i*_DL_ is the capacitive current, *C*_DL_ is the specific capacitance in the non-Faradic region, and *ν* is the scan rate. Electrochemical capacitance was estimated for each sample at the various scan rates, and ECSA was calculated from

**Figure 4:**
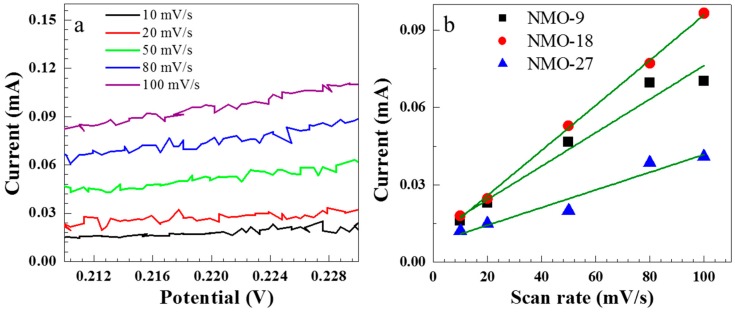
(**a**) Cyclic voltammograms (CV) for NMO-18 at various scan rates in the non-Faradic voltage region (0.21–0.23 V); (**b**) The measured current at 0.22 V as a function of the scan rate for NMO-9, NMO-18, and NMO-27.

ECSA = *C*_DL_/*C*_s_ (2)

where *C*_s_ is the specific capacitance of the standard electrode in an alkaline electrolyte [25]. We chose the non-Faradic region as 0.21–0.23 V, as shown in Figure 4a. Figure 4b shows the *i*_DL_ at 0.22 V for the scan rate, with a corresponding ECSA of 649, 874, and 342 cm^−2^ for NMO-9, NMO-18, and NMO-27, respectively. NMO-18 exhibited the highest ECSA, consistent with its observed maximum supercapacitance. Thus, hydrothermal growth duration is critical to obtain the largest surface area and, hence, the highest supercapacitance.

**Figure 5:**
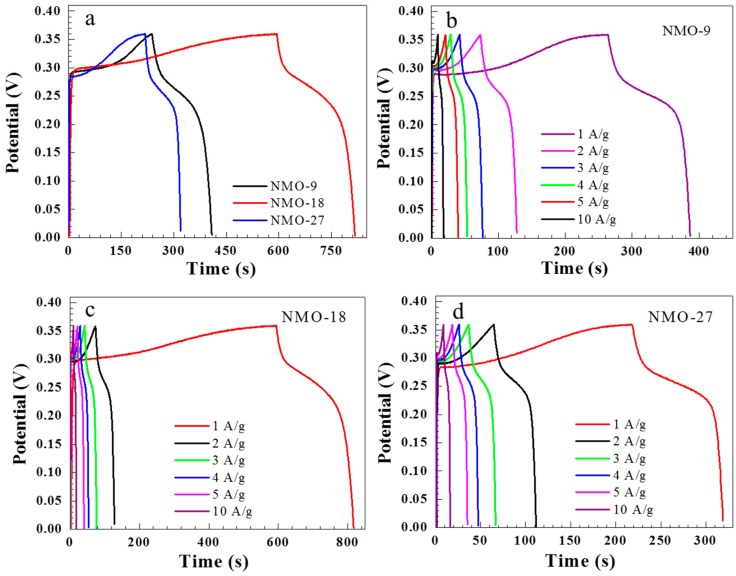
Galvanostatic charge-discharge profile for (**a**) NMO-9, NMO-18, and NMO-27 at 1 A/g current density; (**b**) NMO-9; (**c**) NMO-18; and (**d**) NMO-27 at various current densities.

Figure 5a shows the galvanostatic charge-discharge profiles for the NiMoO_4_ nanostructure electrodes at a 1 A/g current density, with NMO-18 exhibiting the longest discharge time. Figure 5b–d show NMO-9, NMO-18, and NMO-27 charge-discharge profiles, respectively, at different current densities.

**Figure 6:**
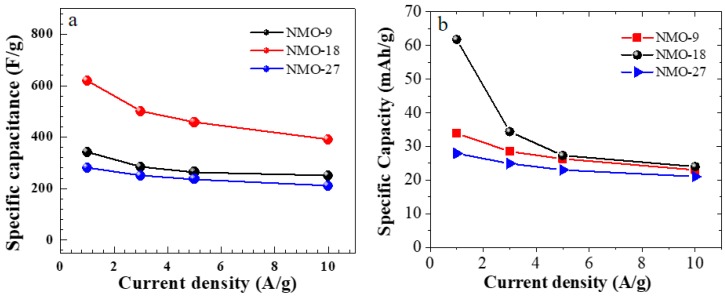
(**a**) Specific capacitance as a function of current density for all the samples of NMO-9, NMO-18, and NMO-27; (**b**) The response of specific capacity to current density for NMO-9, NMO-18, and NMO-27.

Each charge-discharge curve shows pseudo-capacitor characteristics, consistent with the CV data. There is a sharp voltage drop as the supercapacitor changes state from charge to discharge, which can be attributed to an IR drop. Each electrode exhibits nonlinear discharge followed by a plateau, which verifies faradaic reactions occurring on the electrode surface due to the redox reaction at the material/solution interface. Figure 6a represents the specific capacitance response for current densities for all samples. The performance of the specific capacitance decreases with increasing current density. The fall in the supercapacitance value at a high current density could be attributed to the lack of active material at the electrode/electrolyte interface during oxidation/reduction reactions and the slow diffusion of bulky OH ions [26,27].

Specific capacitance or *C*_s_ was calculated from galvanostatic charge-discharge plots (Figure 5) from

(3) $$C_{s}=\frac{I\cdot \Delta t}{m\cdot \Delta V},$$

where *I* is the applied current density, Δ*V* is the potential window, and Δ*t* is the discharge time. The calculated specific capacitances at 1 A/g are 341, 619, and 219 F/g for NMO-9, NMO-18, and NMO-27, respectively. The observed specific capacitances are directly related to the available surface area for the electrochemical reaction in the NiMoO_4_ nanostructure electrode. The superior specific capacitance observed in NMO-18, in comparison with NMO-9 and NMO-27, can be attributed to the higher electrochemical surface area observed in NMO-18 from the ECSA estimation described above. Thus, NiMoO_4_ nanostructures showed comparable capacitance and rate capability with previously reported materials, such as NiO [26,28], Co_3_O_4_ [20], and MnO_2_ [29]. Table 1 shows the super capacitance values of this work along with other NiMoO_4_ reports on various nanostructures and different substrates. The specific capacity was also calculated due to the fact that CV-plots exhibit sharp redox and oxidation peaks arising from a Faradic battery-type mechanism [30,31]. Therefore, we also present specific capacity, *C* (mAh/g), which can be calculated from charge-discharge plots using,

(4) $$C=\frac{I\cdot \Delta t}{3600\cdot m}$$

The specific capacity for all the samples at various current densities are shown in Figure 6b. The specific capacity for NMO-9, NMO-18, and NMO-27 are 33.9, 61.8, and 27.9 mAh/g, respectively, at a current density of 1 A/g.

**Figure 7:**
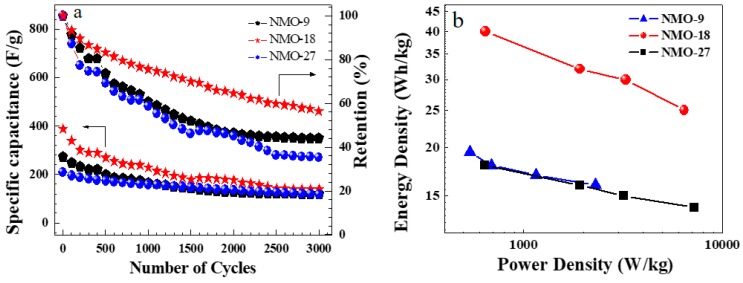
(**a**) Response of specific capacitance over 3000 cycles of charge-discharge operations for NMO-9, NMO-18, and NMO-27; (**b**) Ragone plots with energy density versus power density.

We also study electrochemical stability, an important characteristic for practical applications. Each NiMoO_4_ electrode was subjected to 3000 charge-discharge cycles. Figure 7a shows the electrode stability at 10 A/g current density. NMO-18 exhibited a steep capacitance loss during the initial several hundred cycles, and then stabilized to approximately 57% retention after 3000 cycles [32]. NMO-9 and NMO-27 showed 44% and 56.5% retention after 3000 cycles of charge-discharge operations, respectively. The lower retention level in NMO-9 may be attributed to a more rapid attrition of electrode material in relation to other two samples during charge-discharge cycles. The capacitance reduction can be explained by NiMoO_4_ material physical expansion as ionic transfer occurred and the partial dissolution of NiMoO_4_ material during charge-discharge operations [33]. 

Energy density (*E*) and power density (*P*) can be expressed as

(5) $$E=\frac{1}{2}C_{S}\cdot {\left(\Delta V\right)}^{2}$$

and

(6) $$P=\frac{E}{\Delta t}$$

Figure 7b shows the Ragone plots comparing energy and power density for all the electrodes. The peak energy density was observed for NMO-18 as 40 Wh/kg, and the highest power density of 7200 W/kg was associated with NMO-27. In a largely diffusion-controlled redox reaction between NiO and OH^−^, it is expected that energy density and charge-discharge rates will be inversely proportional [34,35,36]. It is notable that the NiMoO_4_ nanostructures exhibited high power density and energy density, consistent with past research reports [37,38]. Table 2 shows the specific capacitance and energy density found in the current work in relation to the previously published metal oxide reports.

## 4. Conclusions

Direct NiMoO_4_ nanostructure growth on SS substrates was achieved via a facile, single-step hydrothermal method, and the resultant electrode electrochemical characteristics were investigated with respect to growth times of 9, 18, and 27 h. The NMO-18 electrode exhibited the maximum supercapacitance value of 619, 500, 458, and 390 F/g at 1, 3, 5, and 10 A/g, respectively. All the electrodes showed good stability over 3000 charge-discharge cycles. The available active surface area for all the samples were estimated from ECSA analysis. The highest capacitance observed in NMO-18 could be attributed to the observed maximum ECSA value of the sample. Along with excellent capacitive characteristics, NiMoO_4_ showed appreciable energy and power density values. We propose that the direct growth of nanostructured NiMoO_4_ on SS substrate through a one-step hydrothermal method offers a promising electrode material for supercapacitor applications.

## Figures and Tables

**Table 1:** Data of previous NiMoO_4_ nanostructure-based reports compared with our work.

S. No.	NiMoO_4_ Electrode Type	Substrate	Substrate Type	Super Capacitance (F/g)	Reference
**1.**	Nanorods	Ni-Foam	Reactive	1136	[16]
**2.**	Nanorods	Ni-Foam	Reactive	944	[17]
**3.**	Nanotubes	Ni-Foam	Reactive	864	[18]
**4.**	Nanoneedles	Carbon	Non-reactive	412	[19]
**5.**	Nanograins	Stainless Steel	Non-reactive	619	This work

**Table 2:** Electrochemical performance of this work compared with previously reported various metal oxide supercapacitor electrode materials.

S. No.	Electrode Material	Substrate	Specific Capacitance (F/g)	Energy Density (Wh/kg)	Reference
1.	PANI/PTSa/cross linked NiMoO_4_	Graphite	1300	60	[39]
2.	NiMoO_4_ CoMoO_4_ MnMoO_4_	Carbon	412 240 120	35 15 10	[19]
3.	CoMoO_4_	Carbon	79	21	[40]
4.	NiMn_2_O_4_/CNT	Ni	151	60.69	[41]
5.	RuO_2_	-	658	-	[42]
6.	MnO_2_	-	1380	-	[43]
7.	NiMoO_4_	SS	619	40	This work
